# Comparison of High-Normal Versus Low-Normal Mean Arterial Pressure at Target on Outcomes in Sepsis or Shock Patients: A Meta-Analysis of Randomized Control Trials

**DOI:** 10.7759/cureus.52258

**Published:** 2024-01-14

**Authors:** Mohammedsefa A Dari, Azrung Fayaz, Shazia Sharif, Servando Hernandez Galaviz, Eruviel Hernandez Galaviz, Sohaib M Bataineh, Calvin R Wei, Danish Allahwala

**Affiliations:** 1 Otolaryngology - Head and Neck Surgery, Addis Ababa University, Addis Ababa, ETH; 2 Internal Medicine, College of Physicians and Surgeons, Peshawar, PAK; 3 Internal Medicine, Hayatabad Medical Complex Peshawar, Peshawar, PAK; 4 Gastroenterology, Lahore General Hospital, Lahore, PAK; 5 Internal Medicine, Universidad de Monterrey, San Pedro Garza García, MEX; 6 Medicine, Yarmouk University, Irbid, JOR; 7 Research and Development, Shing Huei Group, Taipei, TWN; 8 Nephrology, Fatima Memorial Hospital, Karachi, PAK

**Keywords:** systematic review and meta-analysis, mortality, sepsis, low map, high map

## Abstract

The objective of this meta-analysis was to compare the impact of a high-normal and a low-normal mean arterial pressure (MAP) target on outcomes in patients with sepsis or shock. Adhering to the Preferred Reporting Items for Systematic Reviews and Meta-Analyses (PRISMA) 2020 guidelines, two investigators conducted a thorough literature search across online databases, including PubMed, Cochrane Library, Web of Science, and EMBASE, spanning from inception to December 10, 2023. The assessed outcomes encompassed all-cause mortality, the need for renal replacement therapy, and the length of intensive care unit (ICU) stay. A total of four randomized controlled trials (RCTs) were included, involving 3507 participants with individual study participant counts ranging from 118 to 2463. The pooled analysis revealed no statistically significant difference in the risk of all-cause mortality between the two groups (Risk Ratio (RR): 0.94, 95% Confidence Interval (CI): 0.87 to 1.01). Furthermore, there was no disparity in the rates of renal replacement therapy and the duration of ICU stay between the high-normal and low-normal MAP groups. Our findings indicate no significant distinctions in mortality, rates of renal replacement therapy, or ICU stay duration between the two groups. However, future trials with larger sample sizes are warranted to comprehensively understand the nuanced effects of different MAP settings on outcomes in patients with sepsis and shock.

## Introduction and background

Sepsis has emerged as the primary cause of infection-related fatalities worldwide, affecting nearly 20 million individuals annually, resulting in over 10,000 daily deaths [1,2]. Notably, sepsis-related deaths surpass those from prostate cancer, breast cancer, and AIDS combined as per the data released by the Global Sepsis Alliance [3]. The urgency for decisive progress in sepsis treatment intensifies, as morbidity rates in developed countries have risen by approximately 10% annually over the past decade, posing a cumulative global burden [4]. In developing nations, sepsis mortality persists due to socio-economic challenges and the scarcity of comprehensive epidemiological surveys [5].

The prevailing guidelines for managing sepsis recommend maintaining a mean arterial pressure (MAP) of 65 mm Hg, emphasizing that targeting a higher MAP in septic shock patients offers no discernible benefits [6]. Numerous studies indicate that a MAP exceeding 70 mm Hg may prevent acute kidney injury and enhance microcirculation during septic shock [7-8]. Hemodynamic management is integral to the overall clinical approach in sepsis patients [9], playing a crucial role in stabilizing vital signs early on, impending disease progression, and preventing further tissue and organ failure [10]. However, sustaining a high MAP may necessitate elevated doses of vasoactive drugs, potentially leading to future complications and re-injury to the body [11].

Two previous systematic reviews and meta-analyses aimed to ascertain if higher mean arterial pressure (MAP) targets impact sepsis patient mortality [12-13]. However, recent randomized controlled trials (RCTs) have emerged post these reviews, addressing this clinical question. Consequently, we initiated an updated systematic review and meta-analysis of RCTs, seeking to determine a comprehensive pooled estimate of comparison of effect of a high-normal and a low-normal MAP target on outcomes in sepsis or shock patients.

## Review

Methodology

This meta-analysis followed the Preferred Reporting Items for Systematic Reviews and Meta-Analyses (PRISMA) 2020 guidelines.

Literature Search and Study Selection

Two investigators performed a comprehensive literature search in online databases including PubMed, Cochrane Library, Web of Science, and EMBASE from inception to December 10, 2023. No language restrictions were applied. Keywords used to search for relevant articles were: “Mean atrial pressure”, “sepsis”, "shock", “blood pressure”, “higher” and “lower”. Our search strategy used a combination of medical subject heading (MeSH) terms and synonyms of aforementioned key terms. Additionally, bibliographic lists of all included studies were manually screened to identify additional studies relevant to the study topic.

All relevant research papers were transferred to the EndNote X9 software application, with the manual elimination of duplicate entries. Subsequently, two authors conducted individual assessments of abstracts and titles to identify potentially relevant studies for inclusion. The selected studies underwent a comprehensive full-text review, carried out independently by two investigators, with any disagreements resolved by a third investigator.

Articles were included if they fulfilled the following inclusion criteria: Randomized-control trials of adult human subjects comparing high and low MAP in patients with sepsis and septic shock. Studies reported at least one outcome of interest. A high-normal MAP was defined as MAP of 65 mm Hg or more, while a low-normal MAP was defined as MAP of 60-65 mm Hg. The studies had to maintain the MAP targets for at least 24 hr. We excluded non-randomized studies, reviews, editorials and letters to editors. We also excluded studies that included patients other than sepsis or septic shock.

Data Extraction and Quality Assessment

Two authors independently extracted the following data of interest using a Microsoft Excel spreadsheet: first author, year of publication, country, sample size and characteristics of participants (age, sex, diabetes and hypertension) and clinical outcomes. Outcomes assessed in this meta-analysis include all-cause mortality, the need of renal replacement therapy and length of intensive care unit (ICU) stay.

The evaluation of the included studies' quality was conducted utilizing the Cochrane risk of bias assessment tool, which examines crucial domains like random sequence generation, blinding, and selective reporting to ensure the strength and credibility of the research findings. Two authors independently carried out the quality assessment, and any discrepancies between them were resolved through consensus or discussion involving the third author.

Data Analysis

We conducted the analysis using RevMan version 5.4.1. The summary measure of association for outcomes was represented by the risk ratio (RR). Employing the random-effects method, we calculated the pooled RR across studies, along with corresponding 95% confidence intervals (CIs). Heterogeneity was evaluated using I2 statistics and 95% CIs. Statistical significance was defined as a P value of 0.05 or lower, and heterogeneity was considered significant if I2 exceeded 50%. Forest plots were generated to visually depict the relative effect size of high mean arterial pressure (MAP) goals compared to standard MAP goals for individual clinical endpoints.

Results

**Figure 1:**
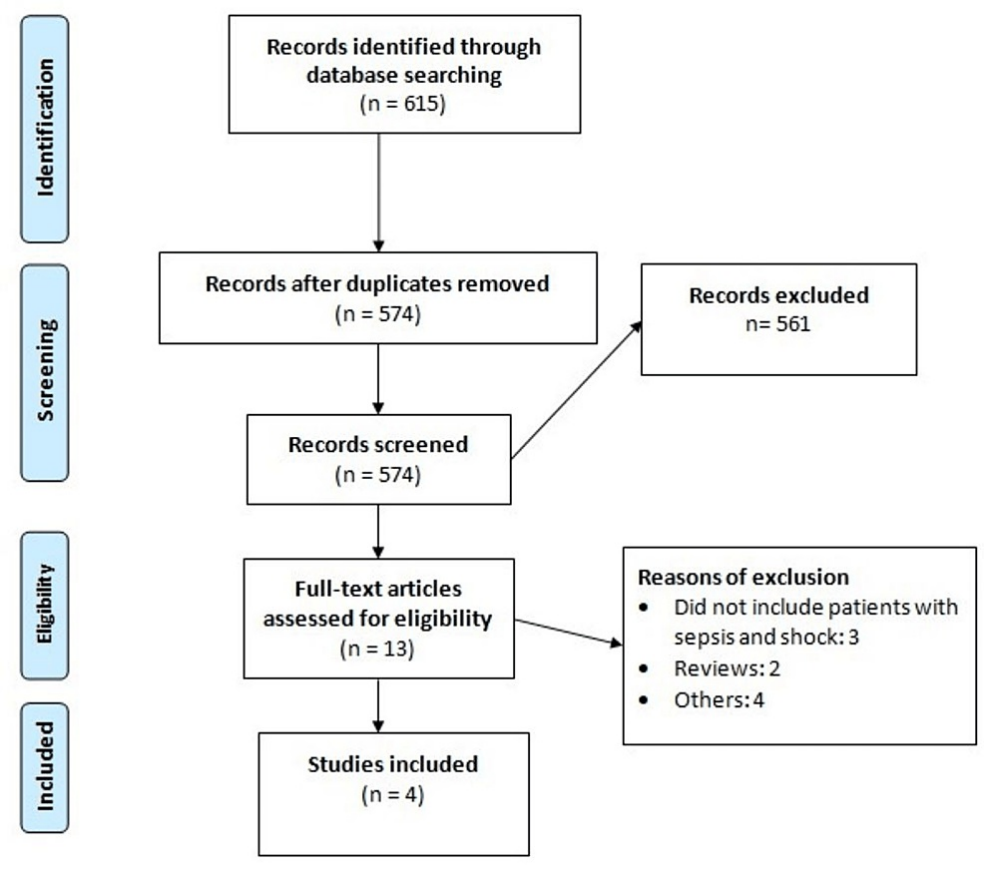
PRISMA chart of study selection process

**Figure 2:**
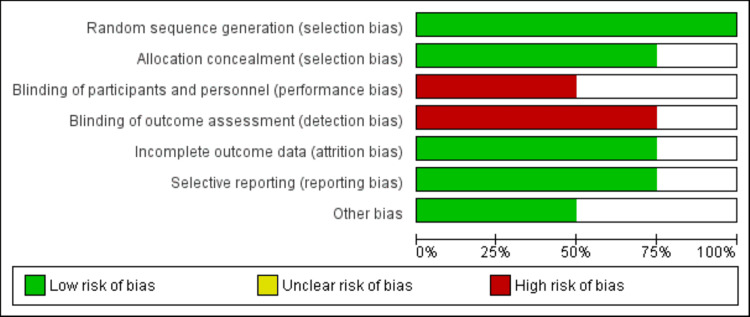
Risk of bias graph

Figure 1 summarizes the study selection process. Electronic databases provided 615 records, and after eliminating duplicates, we assessed 574 studies based on abstracts and titles. Among these, 13 studies underwent a full-text review. In the end, our analysis included four randomized controlled trials (RCTs). The total number of participants across all studies was 3507, with individual study participant counts ranging from 118 to 2463. The characteristics of the studies are presented in Table 1, and the risk of bias graph for the included studies is depicted in Figure 2.

**Table 1:** Study Characteristics MAP: Mean arterial pressure

Author	Year	Region	Groups	Sample Size	Follow-up	Age (Years)	Male (n)	Diabetes (n)	Hypertension (n)
Asfar et al. [14]	2014	France	Low MAP	388	90 Days	65	250	90	NR
			High MAP	388		65	267	75	NR
Lamontagne et al. [15]	2016	Multicenter	Low MAP	60	180 Days	66	31	NR	34
			High MAP	58		63	33	NR	19
Lamontagne et al. [16]	2020	Multicenter	Low MAP	1221	90 Days	75.2	696	19	590
			High MAP	1242		74.8	692	34	597
Maiwall et al. [17]	2023	India	Low MAP	75	28 Days	46.7	65	18	7
			High MAP	75		45	68	14	7

All-cause Mortality

**Figure 3:**
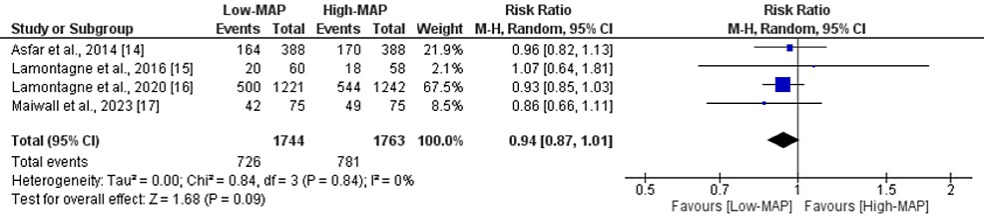
Comparison of effect of low-MAP versus high-MAP on all-cause mortality MAP: Mean arterial pressure Sources: References [14-17]

All four studies included in the analysis reported all-cause mortality, and Figure 3 displays the forest plot. The combined analysis indicated no significant difference in the risk of all-cause mortality between the two groups (Risk Ratio (RR): 0.94, 95% Confidence Interval (CI): 0.87 to 1.01). There was negligible statistical heterogeneity between the studies (I-square: 0%). We conducted a leave-one-out meta-analysis, and the results are detailed in Table 2. As indicated in Table 2, the leave-one-out meta-analysis did not result in any substantial alteration to the final point-estimate.

**Table 2:** Sensitivity analysis (by removing one study at a time) RR: Risk ratio; CI: Confidence interval

Study Id	RR (95% CI)	I-square
Asfar et al., 2014 [14]	0.93 (0.85-1.01)	0%
Lamontagne et al., 2016 [15]	0.93 (0.87-1.01)	0%
Lamontagne et al., 2020 [16]	0.94 (0.82-1.08)	0%
Maiwall et al., 2023 [17]	0.94 (0.87-1.02)	0%

Renal Replacement Therapy

**Figure 4:**
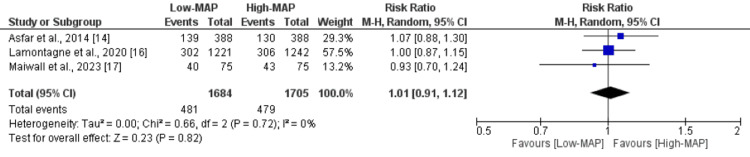
Comparison of effect of low-MAP versus high-MAP on renal replacement therapy MAP: Mean arterial pressure Sources: References [14, 16-17]

Rates of renal replacement therapy were reported in three studies, and Figure 4 illustrates the forest plot. The analysis indicated no discernible difference in the rates of renal replacement therapy between the high-normal and low-normal mean arterial pressure (MAP) groups (Risk Ratio (RR): 1.01, 95% Confidence Interval (CI): 0.91 to 1.12). There was no notable heterogeneity among the study results (I-square: 0%).

Length of ICU Stay

**Figure 5:**
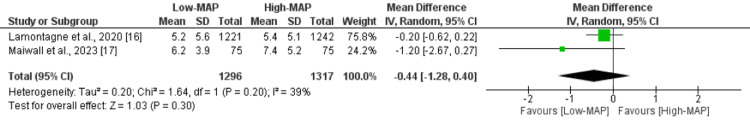
Comparison of the effect of low-MAP versus high-MAP on length of stay in ICU MAP: Mean arterial pressure Sources: References [16-17]

Two studies compared the length of ICU stay between two groups and the forest plot is shown in Figure 5. There was no significant difference in the mean length of ICU stay between the high-normal and low-normal MAP groups (MD: -0.44, 95% CI: -1.28 to 0.40). No significant heterogeneity was reported among the study results (I-square: 39%).

Discussion

In this meta-analysis of four RCTS enrolling patients with sepsis and septic shock comparing high-normal and low-normal MAP groups, there was no significant difference in mortality, renal replacement therapy and duration of ICU stay.

An earlier meta-analysis, combining data from two RCTs on septic shock, revealed no enhancement in the overall 28-day survival in the higher MAP group. Notably, it indicated elevated mortality in elderly patients exposed to vasopressors for over 6 hours [18]. In our analysis, we did not observe heightened mortality associated with a high MAP goal; however, there was a numerical increase in mortality in the high MAP group, which did not reach statistical significance. Unfortunately, we were unable to conduct an age-related analysis due to insufficient data availability.

Our results are consistent with a recent meta-analysis that reported no difference in mortality (Risk Ratio (RR): 1.06; 95% Confidence Interval (CI): 0.98 to 1.15; based on six randomized controlled trials) or the need for renal replacement therapy (RR: 0.96; 95% CI: 0.83 to 1.11; based on three randomized controlled trials) [19]. Notably, our meta-analysis specifically focused on studies involving sepsis or septic shock patients. Another distinction is our examination of the difference in ICU duration between the two mean arterial pressure (MAP) strategies. Despite finding no advantage in a high-normal MAP strategy, this contrasts with a potential benefit noted in a previous systematic review [20]. It's essential to acknowledge that the prior review exclusively considered patients admitted after cardiac arrest, possibly accounting for the disparate outcomes.

Globally, the Mean Arterial Pressure (MAP) holds a pivotal role in sepsis management, serving as a fundamental determinant of organ perfusion pressure [21]. Simultaneously, intensive care teams emphasize achieving specific Systolic Blood Pressure (SBP) targets to optimize blood pressure and guide vasopressor titration for comprehensive hemodynamic stability [22]. This dual focus on MAP and SBP highlights the multifaceted approach to ensuring effective organ perfusion in critical care scenarios.

The microcirculation is increasingly recognized as a critical endpoint for septic shock resuscitation. Non-invasive devices like sidestream dark field (SDF) imaging and near-infrared spectroscopy facilitate microcirculatory assessment [23]. Observational studies indicate significant alterations in the microcirculation of septic shock patients [24-25]. The impact of increasing MAP on the microcirculation varies across studies that reported this parameter [26]. The reasons for these discrepancies, whether related to measurement tools, patient differences, site of measurement, or a combination thereof, remain unclear. Further investigations are warranted to assess the significance of these measurements and the type of intervention (i.e., increase in flow, pressure, or both). The ultimate goal is to establish a relationship between changes in microcirculatory blood flow and improvements in organ function, and ideally, survival.

Setting a MAP goal is pertinent for septic shock outcomes. While a low-MAP target strategy generally mirrors high-target outcomes [14], a fixed value is not universally applicable. The ideal mean blood pressure target likely spans 65 to 85 mm Hg, with a probable sweet spot between 65 and 75 mm Hg for most patients. High MAP targets may induce adverse effects, such as atrial fibrillation, often attributed to elevated vasopressor doses. Chronic hypertension patients may benefit from a target near 85 mm Hg, showing reduced renal impairment [14]. Supporting this, in an early goal-directed therapy study, Rivers et al. noted MAPs of 95 mm Hg in the early-goal group and 81 mm Hg in the control group six hours post-resuscitation initiation, with around 66% having chronic hypertension [27]. Crucially, the 'optimal' MAP varies among patients and within the same patient over time, necessitating repeated assessments to confirm organ function adequacy at the chosen MAP.

The present meta-analysis has certain limitations. Firstly, only four RCTs were included in this meta-analysis. We need more clinical trials with large sample size to understand the effect of different MAP settings on clinical outcomes in sepsis and septic shock patients. Secondly, we were not able to perform subgroup analysis due to a lack of individual-level data. Therefore, in the future, studies should perform subgroup analyses to understand the impact of MAP settings on different groups on the basis of age, gender and comorbidities. Ongoing trials like the Optimal Vasopressor Titration in patients 65 years and older (OVATION-65 trial) are poised to enhance our understanding by evaluating MAP goals' impact on end-organ biological markers [28].

## Conclusions

In this meta-analysis encompassing four RCTs involving patients with sepsis and shock, comparing high-normal and low-normal MAP groups, we found no significant differences in mortality, rates of renal replacement therapy, or duration of ICU stay. While our analysis contributes valuable insights, additional robust clinical trials with larger sample sizes are imperative for a comprehensive understanding of the nuanced effects of different MAP settings on outcomes in sepsis and shock patients. Our meta-analysis underscores the complexity of MAP management in septic shock, urging clinicians to adopt an individualized and iterative approach to confirm organ function adequacy at the chosen MAP.

## References

[1] Wang C, Chi C, Guo L (2014). Heparin therapy reduces 28-day mortality in adult severe sepsis patients: a systematic review and meta-analysis. Crit Care.

[2] Liu D, Mei L, Zhao P (2021). Immunomodulatory effects of anaesthetic sevoflurane in septic mouse model. Saudi J Biol Sci.

[3] Zhou J, Yang D, Liu K, Hou L, Zhang W (2019). Systematic review and meta-analysis of the protective effect of resveratrol on multiple organ injury induced by sepsis in animal models. Biomed Rep.

[4] Perner A, Gordon AC, De Backer D (2016). Sepsis: frontiers in diagnosis, resuscitation and antibiotic therapy. Intensive Care Med.

[5] Sands K, Carvalho MJ, Portal E (2021). Characterization of antimicrobial-resistant Gram-negative bacteria that cause neonatal sepsis in seven low- and middle-income countries. Nat Microbiol.

[6] Dellinger RP, Levy MM, Rhodes A (2013). Surviving sepsis campaign: international guidelines for management of severe sepsis and septic shock: 2012. Crit Care Med.

[7] Moman RN, Ostby SA, Akhoundi A, Kashyap R, Kashani K (2018). Impact of individualized target mean arterial pressure for septic shock resuscitation on the incidence of acute kidney injury: a retrospective cohort study. Ann Intensive Care.

[8] Xu JY, Ma SQ, Pan C (2015). A high mean arterial pressure target is associated with improved microcirculation in septic shock patients with previous hypertension: a prospective open label study. Crit Care.

[9] García-de-Acilu M, Mesquida J, Gruartmoner G, Ferrer R (2021). Hemodynamic support in septic shock. Curr Opin Anaesthesiol.

[10] Leone M, Asfar P, Radermacher P, Vincent JL, Martin C (2015). Optimizing mean arterial pressure in septic shock: a critical reappraisal of the literature. Crit Care.

[11] Kędziora A, Piątek J, Hymczak H (2021). Early postoperative hemodynamic instability after heart transplantation - incidence and metabolic indicators. BMC Anesthesiol.

[12] D'Aragon F, Belley-Cote EP, Meade MO (2015). Blood pressure targets for vasopressor therapy: a systematic review. Shock.

[13] Hylands M, Moller MH, Asfar P (2017). A systematic review of vasopressor blood pressure targets in critically ill adults with hypotension. Can J Anaesth.

[14] Asfar P, Meziani F, Hamel JF (2014). High versus low blood-pressure target in patients with septic shock. N Engl J Med.

[15] Lamontagne F, Meade MO, Hébert PC (2016). Higher versus lower blood pressure targets for vasopressor therapy in shock: a multicentre pilot randomized controlled trial. Intensive Care Med.

[16] Lamontagne F, Richards-Belle A, Thomas K (2020). Effect of reduced exposure to vasopressors on 90-day mortality in older critically ill patients with vasodilatory hypotension: a randomized clinical trial. JAMA.

[17] Maiwall R, Rao Pasupuleti SS, Hidam AK (2023). A randomised-controlled trial (TARGET-C) of high vs. low target mean arterial pressure in patients with cirrhosis and septic shock. J Hepatol.

[18] Lamontagne F, Day AG, Meade MO (2018). Pooled analysis of higher versus lower blood pressure targets for vasopressor therapy septic and vasodilatory shock. Intensive Care Med.

[19] Carayannopoulos KL, Pidutti A, Upadhyaya Y (2023). Mean arterial pressure targets and patient-important outcomes in critically ill adults: a systematic review and meta-analysis of randomized trials. Crit Care Med.

[20] Bhate TD, McDonald B, Sekhon MS, Griesdale DE (2015). Association between blood pressure and outcomes in patients after cardiac arrest: a systematic review. Resuscitation.

[21] Boerma EC, Ince C (2010). The role of vasoactive agents in the resuscitation of microvascular perfusion and tissue oxygenation in critically ill patients. Intensive Care Med.

[22] Mouncey PR, Osborn TM, Power GS (2015). Trial of early, goal-directed resuscitation for septic shock. N Engl J Med.

[23] De Backer D, Ospina-Tascon G, Salgado D, Favory R, Creteur J, Vincent JL (2010). Monitoring the microcirculation in the critically ill patient: current methods and future approaches. Intensive Care Med.

[24] Leone M, Blidi S, Antonini F (2009). Oxygen tissue saturation is lower in nonsurvivors than in survivors after early resuscitation of septic shock. Anesthesiology.

[25] Sakr Y, Dubois MJ, De Backer D, Creteur J, Vincent JL (2004). Persistent microcirculatory alterations are associated with organ failure and death in patients with septic shock. Crit Care Med.

[26] Dubin A, Pozo MO, Casabella CA (2009). Increasing arterial blood pressure with norepinephrine does not improve microcirculatory blood flow: a prospective study. Crit Care.

[27] Rivers E, Nguyen B, Havstad S (2001). Early goal-directed therapy in the treatment of severe sepsis and septic shock. N Engl J Med.

[28] Masse MH, Battista MC, Wilcox ME (2020). Optimal VAsopressor TitraTION in patients 65 years and older (OVATION-65): protocol and statistical analysis plan for a randomised clinical trial. BMJ Open.

